# Levels of physical activity in four domains and affective wellbeing before and during the Covid-19 pandemic

**DOI:** 10.1186/s13690-021-00651-y

**Published:** 2021-07-05

**Authors:** Eliane S. Engels, Michael Mutz, Yolanda Demetriou, Anne K. Reimers

**Affiliations:** 1 Friedrich-Alexander-University Erlangen-Nuremberg, Gebbertstr. 123b, 91052 Erlangen, Germany; 2 Justus-Liebig-University Giessen, Kugelberg 62, 35394 Giessen, Germany; 3 Technical University of Munich, Georg-Brauchle-Ring 60/62, 80992 Munich, Germany

**Keywords:** Covid-19 pandemic, Trend data, Domains of physical activity, Affective wellbeing, Gender

## Abstract

**Background:**

Latest studies indicated that the general mental health level is low during the pandemic. Probably, this deterioration of the mental health situation is partly due to declines in physical activity. The aim of this study was to investigate differences in and the association between affective wellbeing and levels of different domains of physical activity at three time points before and during the pandemic.

**Method:**

We used a nationwide online panel with a trend data design encompassing a total sample of *N* = 3517, representing the German population (> 14 years). Four different activity domains (sport and exercise, light outdoor activity, housework/gardening, active travel) and affective wellbeing (positive and negative affect) were assessed at three time points before and during the Covid-19 pandemic (October 2019, March 2020, October 2020).

**Results:**

Multivariate analyses of variance **(**MANOVA) indicate differences regarding affective wellbeing over the three time points with the lowest values at the second time point. Levels of activity in the four domains differed significantly over time with the strongest decrease for sport and exercise from the first to the second time point. Partial correlations indicated that the relationships between sport and exercise and positive affect were most consistent over time.

**Conclusions:**

Overall, our findings suggest that physical activity plays a particularly important role in the pandemic period as a protective factor against poor mental health. Especially sports and exercise seem to be supportive and should be encouraged, e.g. by providing additional support in finding adequate outdoor, home-based or digital substitutes.

## Background

The outbreak of the new Coronavirus (SARS-CoV-2) at the beginning of 2020 has changed the lives of many people worldwide. The measures and restrictions taken by the national governments to stop the pandemic (e.g., closing of schools, public facilities, gyms, sports clubs) have affected the habits and daily routines of many people. Children and adolescents in Germany reduced their organized sports activity during the pandemic, however overall physical activity levels increased due to a substantial increase in levels of unorganized physical activities like playing outside [38]. In adults, a decrease in physical activity levels was observed [4, 32]. This decline is partly due to external restrictions such as closed gyms and sports clubs. Additionally, the increased remote work lead to a reduction of physical activity that comes along with active commuting [32]. Especially in adults, these circumstances reinforced the already existing problem that many people suffer from a lack of physical activity: Even before the pandemic, only 35% of women and 43% of men in Germany achieved the physical activity recommendations of the World Health Organization [3], according to which adults should either be moderately physically active for 150 min a week or intensively physically active for 75 min [22].

Latest studies indicated that the general mental health level is low during the pandemic [40]. Probably, this deterioration of the mental health situation is partly due to declines in physical activity [20], because regular and maintained participation in physical activity is associated with positive mental health: a number of studies have shown that regular and maintained participation in physical activity is associated with positive mental health [39] such as mental wellbeing [43], happiness [10] and life satisfaction [2, 33]. These positive correlations were found in youths [29], adults [11, 27], and elderly [24]. It is also well examined that inactivity has negative effects on mental health such as an increase in stress [41] and depression [26]. Studies have shown that in people who are usually regularly physically active, even a short period of 1 week of inactivity is enough to significantly increase negative mood [12].

Only few studies have investigated whether there are differences between activity domains and their effects on affective wellbeing. Physical activities in leisure time seem to have more positive effects on wellbeing than activities in work- or transport-related domains [16, 42]. These differential effects may result from the higher level of perceived autonomy and self-determination that are usually associated with leisure time sports activities [10, 11]. Another study showed that active travel was associated with better affective wellbeing in physically highly active and moderately active individuals [28]. Especially for the physically inactive individuals, household or work-related physical activity was found to improve affective wellbeing [28].

Presumably, physical activity plays a particularly important role in the pandemic period as a protective factor against poor mental health. For example, a study conducted in Spain showed that individuals who regularly engaged in intensive physical activity reported higher resilience during self-isolation at home [5]. Moreover, negative associations between moderate-to-vigorous-physical activity per day in hours and poor mental health during the pandemic were found [20]. However, whether or not light physical activity positively influences mental health during the pandemic is not yet clarified [34]. Findings from a cross-sectional survey from Germany indicate that people who reduced sports showed a stronger decline in wellbeing, whereas people who were able to maintain or intensify sports activities reported more positive and less negative affect [31]. A prospective cohort study with students in the UK found that the lockdown measures negatively influenced physical activity behavior and mental wellbeing in students [37].

With regard to socio-demographic aspects, changes in physical activity behavior were stronger in men than in women, which could be due to gender differences in the participation in different domains of activities [37]. In addition, gender differences emerged with regard to the association between regular vigorous physical activity and resilience during the pandemic. Men tended to be more active and resilient than women [5].

Many experts argue that it is important to maintain regular physical activity during the pandemic to protect mental health [8, 15]. Nevertheless, it has not yet been empirically investigated whether different domains of physical activity such as daily activities (e.g., active travel) or sport activities contribute to affective wellbeing during the pandemic in a different way.

Thus, the main purpose of this study was to investigate differences in and the association between affective wellbeing and levels of physical activity in different domains in men and women during the pandemic. For this, we considered different time points of the pandemic (before the pandemic, first lockdown, beginning of second wave) that are associated with different everyday life constraints such as contact restrictions or closure of sport facilities. Specifically, we investigate 1) differences of affective wellbeing and levels of physical activity in four domains in men and women at the three time points, 2) we examine the association between different domains of physical activity and affective wellbeing in men and women at the three time points, and 3) if the three time points of the pandemic moderate the association between the different domains of physical activity behavior and wellbeing. Based on these findings, future recommendations can be developed on which physical activity domains can contribute to a positive affective wellbeing being in men and women.

## Methods

### Procedure and participants

The present study is based on the SPOVID project (Examining physical activity and sports behavior in the face of Covid-19 pandemic: a social inequality perspective) that used a large scale, trend data design with three cross-sectional surveys at different time points: T1 = before the pandemic (from October 18 to November 4, 2019), T2 = during the first lockdown (from March 27 to April 6, 2020) and T3 = beginning of the second wave (from October 16 to November 3, 2020). A nationwide representative sample from Germany was invited via email to participate in the survey. Representativeness was accomplished by integrating the study into an existing nation-wide online panel to which access was provided by Forsa, a leading organization in public opinion polling [13].

The surveys were conducted using computer assisted web interviewing (CAWI) and people were able to answer the survey with a tablet, smartphone or computer. Surveys were designed to be answered in 15 min. Participants were recruited solely offline via telephone surveys, so that people who use the internet sparsely are also adequately represented in the sample. All respondents gave written consent to be contacted for this study and participated voluntarily. The study was approved by the independent Research Ethics Committee of the Medical Faculty of Friedrich-Alexander University Erlangen-Nuremberg, Germany (Ref. No. 387_20B) and conducted in accordance with the 1964 Declaration of Helsinki and its later amendments.

The total sample *N* = 3516 of the study consisted of three independent samples and is representing the German population (> 14 years). The first data collection included *N1* = 1008 participants, the second data collection *N2* = 1001 and third data collection *N3* = 1508. In the T1 sample, younger age groups were intentionally oversampled. Hence, sampling weights were calculated and applied to correct for the disproportionality resulting from the sampling design [35]. All analyses were then performed using the weighted data set. Table 1 shows the sample characteristics regarding to gender and age.

**Table 1:** Sample characteristics regarding gender and age

		***Gender***		***Age***	
	***N***	***Men***	***Women***	***M***	***SD***
Total sample	3516	49.6	50.4	47.88	18.20
Sample (T1)	1008	50.1	49.9	44.96	17.71
Sample (T2)	1001	50.0	50.0	49.38	17.69
Sample (T3)	1507	49.0	51.0	48.85	18.65

*Notes.* T1 = First time point (October 2019); T2 = second time point (April 2020); T3 = third time point (October 2020); weighted data

### Instruments and measures

#### Levels and domains of physical activity

Four domains of activity were measured (independent variables): sport and exercise (such as jogging, playing soccer or fitness training); light outdoor activity (such as hiking or going for walks); housework and gardening; active travel (by foot or by bicycle). For each domain of physical activity we assessed the levels by number of hours per week (“How many hours of your leisure time have you spent in total in the last week with sports and physical activity?”) using an eight-point rating scale. Answers were recoded so that numeric values best represent a persons’ engagement in physical activity in hours per week (0 = “no time at all”, 0.5 = “less than 1 hours”, 1 = “1 h”, 2 = 2 h”, 3.5 = 3 to 4 h”, 5.5 = “5 to 6 hours”, 10.5 = “7 to 14 hours”, and 16 = “15 hours or more”).

#### Affective wellbeing

We assessed affective wellbeing (dependent variable) with two factors (positive affect and negative affect) by three items each, adapted from the European Social Survey Module on ‘Personal and Social Well-being’ [19]. Respondents were asked: “How often have you felt the following states of feelings in the last week? Positive affect (e.g. “I was happy”) and negative affect (e.g., “I was sad”) was measured with a four-point rating scale. Answers were recoded so that numeric values best represent a persons’ indication of frequency in days per week (0 = “not at all”, 2 = “on a few days”, 4 = “more than half of the days”, and 6 = “almost every day”).

#### Socio-demographic factors

We included three socio-demographic factors as potential correlates of physical activity and/or wellbeing, namely age (in years), gender (women vs. men), and educational degree (3 categories from low to high degree).

### Data analysis

All analyses were performed using SPSS (Statistical Package for the Social Sciences) Version 26 (IBM Corporation, Armonk, NY). Internal consistencies for both scales of affective wellbeing (positive and negative) were proved [9]. Descriptive statistics for all variables were calculated for each sample of the three time points, each separately for men and women. We performed multivariate analyses of variance (MANOVA) with planned contrasts to compare mean values of wellbeing and physical activity domains regarding the three time points. As post-hoc corrections of multiple comparisons, we used Bonferroni correction to avoid the accumulation of the alpha error. Moreover, we conducted MANOVA to analyze gender differences regarding wellbeing and physical activity domains. Furthermore, partial correlations were performed to examine relationships between different domains of activity and affective wellbeing, separately for women and men. To analyze, if the relationship between different domains of physical activity and wellbeing is moderated by time (before the pandemic, lockdown, beginning of second wave), we conducted a moderation analysis using *Process macro 3.1* (Model 1) developed by Hayes [17] for each domain of physical activity, separately for women and men. For all calculations a weighting factor was applied to correct age disproportionality resulting from the sampling design [35]. To control for potential covariates, we included age and educational degree in all analyses.

## Results

### Affective wellbeing

Internal consistencies (Cronbach’s alpha) for the two scales to assess wellbeing ranged from acceptable (α = .70) for negative affect to good (α = .82) for positive affect. Scale means and standard deviations for affective wellbeing are reported in Table 2 for the three time points and were calculated separately for men and women. We found the highest scale means regarding positive affect (i.e. “I was happy”) before the beginning of the pandemic (Time 1) for men (*M* = 3.66; *SD* = 1.52). The lowest scale means of positive affect were observed during the first lockdown (Time 2) for women (*M* = 2.75; *SD* = 1.67). Regarding negative affect (i.e. “I was sad”), the descriptive findings showed the highest scale mean before the pandemic (Time 1) for women (*M* = 1.70; *SD* = 1.33). The lowest scale means of negative affect were observed for men during the first lockdown (Time 2) (*M* = 1.25; *SD* = 1.20).

**Table 2:** Differences in positive and negative affect between gender and at the three time points

	***Positive affect***						***Negative affect***					
	***M***	***SD***	***F***	***df***	***p***	***η***_***p***_^***2***^	***M***	***SD***	***F***	***df***	***p***	***η***_***p***_^***2***^
**Time 1**	3.57	1.57					1.55	1.21				
Men	3.66	1.52	2.40	1	.122	.002	1.52	1.13	4.29	1	.039	.004
Women	3.50	1.61					1.61	1.28				
**Time 2**	2.90	1.66					1.43	1.31				
Men	3.06	1.63	8.41	1	.004	.008	1.25	1.20	19.01	1	.001	.019
Women	2.75	1.67					1.60	1.38				
**Time 3**	3.38	1.63					1.53	1.30				
Men	3.48	1.60	4.89	1	.027	.003	1.32	1.24	37.82	1	.001	.022
Women	3.28	1.67					1.70	1.33				

*Notes.* Time 1 (October 2019) = before the pandemic; Time 2 (April 2020) = first lockdown; Time 3 (October 2020) = beginning of second wave; *F* = F-value, *df* = degrees of freedom; *p* = level of significance; control variables: age and educational degree

#### Differences over time in affective wellbeing

Findings of the MANOVA showed that affective wellbeing differed significantly between the three time points (before the pandemic, first lockdown, beginning of second wave). We found the highest values for positive affect before the pandemic (Time 1), followed by beginning of second wave (Time 3) and the lowest values at the first lockdown (Time 2) with significant differences (*F*(2) = 46.56, *p* < .001, *η*_*p*_^*2*^ ***=*** .025). We found the same pattern regarding negative affect with the highest values before the pandemic (Time 1), followed by beginning of second wave (Time 3) and the lowest values at the first lockdown (Time 2) with significant differences (*F*(2) = 2.66, *p* = .070, *η*_*p*_^*2*^ ***=*** .001).

#### Gender differences in affective wellbeing

Findings did not show significant gender differences regarding positive affect at the first time point. At the second and the third time point we observed significant slight differences between men and women in terms of positive affect with higher values for men (see Table 2). Regarding negative affect, findings showed significant higher scale means for women than for men over all three time points.

### Domains of physical activity

Means and standard deviations for the different domains of physical activities (sport and exercise, light outdoor activity, housework/gardening and active travel) are reported in Table 3, separately for men and women and the three time points. Considering the different time points, we found for *sport and exercise* the highest mean for men at the first time point (*M* = 2.51; *SD* = 2.96) and the lowest mean at the second time point (*M* = 1.12; *SD* = 2.33). Regarding *light outdoor activity*, we observed the highest mean for women at the first time point (*M* = 3.66; *SD* = 3.95) and the lowest value for men at the third time point (*M* = 3.13; *SD* = 3.62). Considering *housework/gardening*, the highest mean was found for women at the first point (*M* = 4.87; *SD* = 4.52) and the lowest mean for men at the third time point (*M* = 3.28; *SD* = 4.13). Regarding *active travel*, we found the highest mean for men at the first time point (*M* = 2.90; *SD* = 3.41) and the lowest value for women at the third time point (*M* = 2.06; *SD* = 2.74).

**Table 3:** Differences in levels of activity domains between gender and at the three time points

	***Sport and exercise***						***Light outdoor activity***						***Housework/gardening***						***Active travel***					
	***M***	***SD***	***F***	***df***	***p***	***η***_***p***_^***2***^	***M***	***SD***	***F***	***df***	***p***	***η***_***p***_^***2***^	***M***	***SD***	***F***	***df***	***p***	***η***_***p***_^***2***^	***M***	***SD***	***F***	***df***	***p***	***η***_***p***_^***2***^
**Time 1**	2.13	2.72					3.47	3.74					4.31	4.31					2.88	3.50				
Men	2.51	2.96	18.49	1	.001	.019	3.29	3.53	1.24	1	.266	.001	3.82	4.07	9.33	1	.002	.010	2.90	3.41	0.44	1	.508	.000
Women	1.67	2.27					3.66	3.95					4.87	4.52					2.84	3.58				
**Time 2**	1.14	2.33					3.52	3.89					4.48	4.70					2.16	3.10				
Men	1.12	2.33	1.24	1	.725	.000	3.48	3.90	0.56	1	.813	.000	4.31	4.75	1.22	1	.269	.001	2.16	2.97	0.00	1	.991	.000
Women	1.16	2.33					3.54	3.88					4.65	4.64					2.16	3.22				
**Time 3**	1.70	2.68					3.19	3.63					3.57	4.19					2.27	3.00				
Men	1.94	2.99	10.70	1	.001	.006	3.13	3.62	0.42	1	.517	.000	3.28	4.13	4.90	1	.027	.003	2.48	3.20	7.09	1	.008	.004
Women	1.46	2.29					3.23	3.62					3.83	4.11					2.06	2.74				

*Notes.* Time 1 (October 2019) = before the pandemic; Time 2 (April 2020) = first lockdown; Time 3 (October 2020) = beginning of second wave; *F* = F-value, *df* = degrees of freedom; *p* = level of significance; control variables: age and educational degree

#### Differences over time in domains of physical activity

We found significant mean differences for the levels of *sport and exercise* between all three time points (*F*(2) = 35.73, *p* < .001, *η*_*p*_^*2*^ *=* .019) with the lowest levels at the second time point. Findings for *light outdoor activity* showed no significant mean difference (*F*(2) = 3.11, *p* = .045, *η*_*p*_^*2*^ *=* .002) regarding their levels before the pandemic (October 2019), first lockdown (April 2020), and beginning of second wave (October 2020). Regarding *housework/gardening,* mean values were significantly lower at the third time point than at the first and the second time point (*F*(2) = 16.83, *p* < .001, *η*_*p*_^*2*^ *=* .009). For *active travel*, findings showed significant mean differences (*F*(2) = 15.35, *p* < .001, *η*_*p*_^*2*^ *=* .008) between October 2019 and April 2020 and between October 2019 and October 2020, but not between April 2020 and October 2020.

#### Gender differences in domains of physical activity

Considering the first examined time point, findings showed significant differences between men and women only regarding their levels of *sport and exercise* with higher levels for men and for *housework/gardening* with higher levels for women (Table 3). Regarding the second time point there were no significant differences between men and women regarding their activity levels in different activity domains. At the third time point**,** we found significant mean differences between men and women regarding levels of all four activity domains except *light outdoor activity*. Levels of *sport and exercise* and *active travel* were higher for men and levels of *housework/gardening* were higher for women.

### Relationships between levels of physical activity domains and affective wellbeing over time

The partial correlations between levels of different physical activity domains and affective wellbeing were calculated separately for men and women at the three time points (Table 4). Findings regarding the first time point showed for positive affect a significant slight positive correlation with *light outdoor activity, housework/gardening* and *active travel* in men. Negative affect did not correlate significantly with any activity domain in men. For women, *sport and exercise* showed a significant positive correlation with positive affect and a significant negative correlation with negative affect. At the second time point, both men and women showed significant correlations between *sport and exercise* and positive affect. Additionally, for men, *housework/gardening* and for women, *light outdoor activity* and *active travel* correlated significantly positively with positive affect. At the third time point, all four physical activity domains correlated slightly with positive affect in men. In women, no significant correlations with *housework/gardening* and *active travel* existed. Negative affect correlated significantly negatively with all activity domains except for *housework/gardening* in men. For women, no significant correlations were observable with negative affect.

**Table 4:** Partial correlations between affective wellbeing and levels of different activity domains, separately for men and women over the three time point

Variable	Positive affect		Negative affect		Sport and exercise		Light outdoor activity		Housework/ gardening	
	Men	Women	Men	Women	Men	Women	Men	Women	Men	Women
**Time 1**										
**Negative affect**	−.523**	−.568**								
**Sport and exercise**	.070	.238**	.012	−.232**						
**Light outdoor activity**	.136**	.035	.045	−.008	.244**	.105*				
**Housework/gardening**	.108*	−.028	.022	.050	−.067	−.026	.154**	.213**		
**Active travel**	.101*	.045	−.024	−.007	.133**	.114*	.278**	.493**	.109*	.131**
**Time 2**										
**Negative affect**	−.485**	−.513**								
**Sport and exercise**	.129**	.134**	−.097*	.000						
**Light outdoor activity**	.061	.102*	−.032	−.063	.065	.184**				
**Housework/gardening**	.207**	.089	−.074	−.035	.082	.061	.148**	.213**		
**Active travel**	.053	.090*	−.069	−.061	.198**	.160**	.344**	.508**	.171**	.138**
**Time 3**										
**Negative affect**	−.488**	−.527**								
**Sport and exercise**	.164**	.082*	−.130**	−.003						
**Light outdoor activity**	.086*	.101**	−.114**	−.026	.159**	.146**				
**Housework/gardening**	.109**	.059	−.044	−.004	.025	−.016	.163**	.168**		
**Active travel**	.168**	.066	−.139**	.045	.283**	.169**	.304**	.445**	.188**	.162**

*Notes.* Included control variables: age and educational degree; ** the correlation is significant at the level *p* < .01 (two-tailed); * the correlation is significant at the level *p* < .05 (two-tailed)

Additionally, we conducted four different moderation analyses for each domain of physical activity to examine if time (before the pandemic, lockdown, beginning of second wave) moderates the relationship between activity levels in different physical activity domains and wellbeing. Findings did not show any significant interactions by time, neither for men nor for women. Therefore, the relationship between different activity domains and wellbeing were not depending on time.

## Discussion

The present study investigated differences in and the association of affective wellbeing and levels of physical activity in different domains during three time points of the pandemic by taking gender into account.

### Affective wellbeing

The key findings indicate that affective wellbeing differed over the three time points (before the pandemic, first lockdown, beginning of second wave). Surprisingly, the lowest values for positive and also for negative affect were found at the second time point indicating a decrease in positive and negative affect during the lockdown in April 2020. This could indicate that people in lockdown have fewer triggers for both positive and negative emotions (e.g., less anger at work, traffic jams, time pressure) and as a result there is a so-called flattening of affect. Here, however, differentiated investigation would be necessary to test these explanations empirically. Findings regarding gender differences showed that men reported a better affective wellbeing compared to women, which is in line with prior research [5]. Carriedo et al. [5] showed that men reported a higher resilience (e.g. higher self-efficacy and higher optimism) than women during the first week of confinement. Our findings suggest that the first lockdown in April 2020 affected men less negatively in terms of wellbeing. At the first time point, there were no differences between men and women in terms of wellbeing, but at the second and third time point, women appear to be more impaired by the pandemic in terms of wellbeing than men. This may indicate that women suffer more from the double burden of household/childcare and work than men, which is even more apparent in times of pandemic [18, 23].

### Domains of physical activity

The investigation of levels of different activity domains showed differences at the three time points. *Sport and exercise* levels differed over all three time points with the lowest levels at the second time point during the pandemic. This finding is in line with previous research [32, 38] and can be explained by the restrictions such as closing of sport clubs and gyms due to the course of the pandemic. There were no changes in positive or negative direction regarding *light outdoor activity* over the three time points. The relative stability of *light outdoor activity* in the pandemic may be due to the fact that there were few restrictions in Germany that were tangent to this domain. Levels regarding *housework/gardening* were significantly lower at the beginning of the second wave in October 2020 compared to before the pandemic and during the first lockdown. However, men showed higher levels of *housework/gardening* activities during the lockdown in April 2020 than at the other time points. This finding could be explained by the increased time spent at home due to the lockdown situation. Regarding *active travel*, the findings during the pandemic (April 2020 and October 2020) showed a decrease in the levels of *active travel* compared to the time before the pandemic in October 2019. This indicates that active travel was still reduced in October 2020, maybe because many people continued to work from home. At the first time point in October 2019 findings showed significant differences between men and women regarding their levels of *sport and exercise* with higher levels for men and with higher levels of *housework/gardening* in women that are in line with previous findings from large-scale surveys of adults in Germany [22, 30]. By the time of the lockdown in April 2020, the gender differences had briefly disappeared. At the third time point, men showed higher levels than women in *sport and exercise* and *active travel*, but women showed higher levels of *housework/gardening*. The findings on gender differences in activity behavior could be explained by the still prevailing traditional gender roles and corresponding social structures in our society such as differences in employment status, income, and the unequal distribution of household responsibilities and childcare [14, 21]. Furthermore, gender-based sport stereotypes affect self-perceptions and activity-related perceptions, and thus, also have an impact on sport and exercise participation and performance [7].

### Relationships between levels of activity domains and affective wellbeing over time

The comparison for men over the three time points showed that especially during the lockdown in April 2020, *sport and exercise* and *housework/gardening* were relevant for men’ wellbeing. At the third time point, findings for men showed over all four activity domains positive correlations with positive affect. This finding suggests that not only *sport and exercise* contribute to affective wellbeing, also *light outdoor activity*, *housework/gardening* and *active travel* are associated with positive affect. Hence, these findings indicate that staying physically active over such a long period of the pandemic is important, regardless of the physical activity domain. For negative affect, only *sport and* exercise showed a negative relationship at the second time point and at the third time point negative correlations with the activity domains were found for *sport and exercise, light outdoor activity* and *active travel*.

Regarding women it is noticeable that positive affect is not related to *housework/gardening* compared to men. This is presumably due to the fact that women show higher levels across all time points, which then no longer contribute positively to wellbeing after a certain level [1]. Another possible explanation would be that the levels of physical activity in the specific domain of *housework/gardening* activities differ between men and women and therefore contribute differently to wellbeing [6]. Further research is needed to investigate these assumptions and underlying mechanisms. For both men and women, a positive correlation between *sport and exercise* and wellbeing was found during the lockdown. It seems that this domain of activity is most appropriate for positively contributing to wellbeing in both men and women during a lockdown period. This could be explained by the observation that sport and exercise as leisure time activity is often more intrinsically motivated, as it is associated with a higher degree of perceived autonomy and experience of enjoyment [10, 11]. In this regard, it has been argued that physical activities must be “de-instrumentalized” to generate pleasurable experiences that contribute to wellbeing [36]. Other studies showed that effects of household-, workplace-, and transport-related physical activity on affective wellbeing were dependent on individual characteristics such as age, sex, weight status, and participation in other domains [6]. Generally, over all time points, findings regarding relationships suggests that the domains of activity considered are relevant for positive affect, but hardly related to negative affect for both genders. According to the findings of our moderator analyses, the relationships between levels of different physical activity domains and wellbeing were not depending on time for both genders. Therefore, the relationships between levels of *sport and exercise, light outdoor activity, housework/gardening* and *active travel* with affective wellbeing were quite stable and not depending on the measured time point.

In general, inter-correlations between the activity domains are positive and have a slight to moderate size. This suggests that people who are active in one domain of activity (e.g., jogging) are likely to be active in several domains of activity (e.g., biking to work). Only *sport and exercise* and *housework/gardening* did not correlate at all.

The strength of our study are the underlying large nationwide representative online surveys that were based on the same measures (of wellbeing and domain-specific physical activities) and that were conducted at three time points before and during the pandemic. However, the findings of our study are limited to self-reported data that may lead to inaccuracies in participant responses regarding physical activity behavior compared to the assessment with accelerometers, pedometers or other device-based measures. Unfortunately, we did not capture information on working conditions or daycare of children and homeschooling before and during the pandemic. This information would have been relevant for the research question at hand. Competing work and family responsibilities may be associated with higher stress levels and lower mental health [25].

## Conclusions

Findings of the present study showed significant differences of affective wellbeing and levels of different physical activity domains before the pandemic, during the first lockdown and at the beginning of the second wave. Findings indicated a decrease in positive and negative affect during the lockdown in April 2020. At the second and third time point, women appear to be more impaired by the pandemic in terms of wellbeing than men. Levels of sport and exercise differed significantly between all three time points with the lowest levels during lockdown in April 2020, when infrastructure was closed. There were no changes over time in positive or negative direction regarding light outdoor activity. Levels regarding housework/gardening were significantly higher during lockdown in April 2020 compared to other time points. Regarding active travel, findings for the time points during the pandemic (April 2020 and October 2020) showed a decrease in levels of active travel compared to October 2019 before beginning of the pandemic. The main differences regarding gender were found at the beginning of the second wave (October 2020): men showed higher levels in the domains sport and exercise and active travel than women and women showed higher levels in housework/gardening. For men, the relationship between housework/gardening and positive affect was the most consistent over time and for women the relationship between sport and exercise and positive affect. Generally, findings suggest that all considered activity domains are relevant for positive affect, but hardly related to negative affect for both genders. Our findings suggest that staying active during the pandemic is important and contributes to wellbeing, especially sport and exercise seems to be supportive. For practical implications, people should be encouraged to stay physically active in a lockdown period. This may imply to provide additional support, making it easier for them finding adequate outdoor, home-based or digital substitutes for organized sport and exercise activities in clubs and gyms.

## Data Availability

The datasets generated and analyzed during the current study are not publicly available.

## Abbreviations

MANOVA: Multivariate analyses of variance
SPSS: Statistical Package for the Social Sciences
